# Using Focus Groups in the Consumer Research Phase of a Social Marketing Program to Promote Moderate-Intensity Physical Activity and Walking Trail Use in Sumter County, South Carolina

**Published:** 2005-12-15

**Authors:** Ericka Burroughs, Lara E Peck, Patricia A Sharpe, Michelle L Granner, Carol A Bryant, Regina Fields

**Affiliations:** Prevention Research Center, Arnold School of Public Health, University of South Carolina; Prevention Research Center, Arnold School of Public Health, University of South Carolina, Columbia, SC; Prevention Research Center, Arnold School of Public Health, University of South Carolina, Columbia, SCa; Prevention Research Center, Arnold School of Public Health, University of South Carolina, Columbia, SC; Dr Granner is now with the School of Public Health, University of Nevada, Reno, NV; College of Public Health Arts and Sciences, University of South Florida, Tampa, Fla; South Carolina Healthy Schools, South Carolina Department of Education, Columbia, SC

## Abstract

**Introduction:**

The use of social marketing approaches in public health practice is increasing. Using marketing concepts such as the "four Ps" (*product*, *price*, *place*, and *promotion*), social marketing borrows from the principles of commercial marketing but promotes beneficial health behaviors. Consumer research is used to segment the population and develop a strategy based on those marketing concepts. In a community-based participatory research study, 17 focus groups were used in consumer research to develop a social marketing program to promote walking and other moderate-intensity physical activities.

**Methods:**

Two phases of focus groups were conducted. Phase 1 groups, which included both men and women, were asked to respond to questions that would guide the development of a social marketing program based on social marketing concepts. Phase 1 also determined the intervention's target audience, which was irregularly active women aged 35 to 54. Phase 2 groups, composed of members of the target audience, were asked to further define the product and discuss specific promotion strategies.

**Results:**

Phase 1 participants determined that the program product, or target behavior, should be walking. In addition, they identified price, place, and promotion strategies. Phase 2 participants determined that moderate-intensity physical activity is best promoted using the term *exercise* and offered suggestions for marketing walking, or exercise, to the target audience.

**Conclusion:**

There have been few published studies of social marketing campaigns to promote physical activity. In this study, focus groups were key to understanding the target audience in a way that would not have been accomplished with quantitative data alone. The group discussions generated important insights into values and motivations that affect consumers' decisions to adopt a product or behavior. The focus group results guided the development of a social marketing program to promote physical activity in the target audience in Sumter County, South Carolina.

## Introduction

In a community-based participatory research study funded by the Centers for Disease Control and Prevention (CDC), researchers worked with a community advisory council to design a social marketing program promoting moderate-intensity physical activity (specifically walking and trail use) in Sumter County, South Carolina. The county has 104,000 residents and is one of many rural counties located in South Carolina. The population is 50.1% white, 46.7% black, and 3.2% other racial or ethnic groups, mainly Hispanic (1).

In 2003, consumer research was conducted by analyzing a year of quantitative and qualitative data. Quantitative data were collected with a countywide random-digit–dialed telephone survey (E.L.B., unpublished data, 2003). Qualitative data were collected through focus groups; this article describes the qualitative findings and illustrates the value of using focus groups as a consumer research tool. 

Qualitative data, such as those collected in focus groups, provide in-depth information necessary to understanding attitudes and motivations that influence consumers' decisions and behavior. Focus groups give participants an opportunity to describe their experiences and preferences without the limitation of preset response categories (2). Focus groups also are helpful because researchers want participants to interact with each other, sharing ideas in their own words and responding to each other's comments.

Social marketing is a strategy that employs principles of commercial marketing to influence consumer behavior or decision making. It represents a set of principles comprising an approach to health promotion but not a theory of behavioral change; therefore, social marketing campaigns often include other behavioral-change strategies, such as enhancing social support and promoting behavioral skills. Social marketing was selected to promote physical activity across the county because it can influence individual-level behavior change through community-wide interventions.

A key feature of social marketing is its consumer-centered approach, meaning that the consumers' perceived needs, values, and preferences are considered before a behavior modification strategy is determined and promoted. Variables known as the "four Ps" (*product, price, place,* and *promotion*) influence consumers' decisions to change their behaviors (3). Consumer research is the first step to assessing consumer preferences for and opinions about these variables.

In social marketing,* product* refers to the behavior being promoted and its associated benefits. As Kotler et al (4) note, marketers distinguish between the actual product (the behavior being promoted) and the core product (the benefits of the desired behavior). Because competition is an essential component of marketing, social marketing programs attempt to identify the benefits that best distinguish the product from its competition (5). *Positioning* refers to how the product will be aligned relative to competing demands in consumers' minds. To increase demand for the product, it must be positioned to minimize competition from these competing demands. For example, some women may attend to family matters before caring for themselves, or they may choose to forgo physical activity to watch their favorite television show.

In social marketing, *price* is best conceptualized as the costs people must exchange for product benefits. Price includes emotional, social, and psychological costs in addition to monetary exchanges and includes barriers to adopting or purchasing a behavior or product. According to exchange theory, if the consumer perceives that the price is too high, the product is not likely to be purchased. Conversely, if the consumer feels a product is affordable and the costs are outweighed by the benefits, the product is likely to be purchased. As stated by McCormack, "The objective of the pricing strategy is to lower the perceived costs and/or make them more acceptable to potential consumers" (3). 

*Place* can refer to the ideal location where consumers can obtain information about the product (3). In this study, place refers to when and where consumers can practice the product or behavior, with the goal of making it more accessible (5). With walking, the place can range from a treadmill at home to a local gym or a trail. If consumers are dissatisfied with the places where the behavior can be practiced, they are less likely to adopt it on a regular basis. Because there are several new walking trails and paths in Sumter County that are not often used, the community advisory council and the local county recreation department wanted to increase trail use in the county.

*Promotion* involves a carefully designed set of activities intended to influence change and usually includes a wide range of activities to create demand for the product (6). The promotional strategy highlights the benefits of the product while minimizing the price and offering attractive places where the consumer may gain access to the product. Promotion also includes any outreach, media, and incentives that would be used to market the product to the consumer (5). 

Social marketing has been used successfully to increase breast cancer screening (3) and breastfeeding rates (7). *The Guide to Community Preventive Services* (8), a comprehensive review of the effectiveness of interventions to increase physical activity, found strong evidence for community-wide campaigns and social-support interventions.  

Insufficient evidence is available to assess the efficacy of mass media campaigns (8); however, mass media interventions have been used to promote physical activity (9,10). All three of these approaches — community-wide campaigns, social-support interventions, and mass media campaigns — may be incorporated into a social marketing campaign; however, there have been few published studies of social marketing campaigns to promote physical activity (11,12).

## Methods

Focus groups were conducted in two phases. All participants resided in Sumter County, South Carolina. The first phase of the focus groups included diverse members of the community, both physically active and inactive, to characterize the opinions on and preferences for physical activity promotion at the broadest level and to identify and characterize community subgroups by age, sex, race or ethnicity, activity level, and socioeconomic status (SES). This initial characterization guided a tailored marketing approach and selection of the target audience. 

The University of South Carolina Institutional Review Board approved the study procedures. For each set of focus groups, a project coordinator used a purposive convenience sampling approach (13,14), an approach in which participants are selected based on certain characteristics. For this study, these characteristics included age, sex, race or ethnicity, activity level, and SES. The project coordinator first recruited community members who then volunteered to recruit group participants by word of mouth and by distributing flyers about the focus groups in their social and occupational circles. The project coordinator then screened potential participants to determine whether they met the parameters for the focus group and to ensure each group was as homogeneous as possible. Before each group discussion, participants were informed about the purpose of the project and provided an informed consent agreement. Each was offered a $20.00 money order to offset any costs, such as transportation or childcare, associated with participating in the group.

Each group discussion was held at a local community center, a local church, or the county recreation and parks department. They were moderated by an experienced interviewer, audiotaped, and transcribed verbatim by a professional transcriptionist. Additionally, a recorder took notes to assist with accurate transcription.

### Phase 1

Twelve groups varying in age, race, and activity level were selected for Phase 1 (Table 1). There were 27 men and 63 women for a total of 90 participants. Before the discussion, participants completed an anonymous questionnaire on their demographic characteristics. Fifty seven (63.0%) of the participants were black, 30 (33.0%) were white, 2 (2.2%) were Hispanic or Asian, and 1 (1.1%) was unidentified. The majority of the groups included irregularly active participants. Irregularly active was defined as participating in some physical activity but not enough to meet current physical activity recommendations of 30 minutes of moderate-intensity physical activity per day on 5 or more days of the week or 20 minutes of vigorous activity on 3 or more days per week (15,16). One group of men and one group of women interviewed, however, did meet the recommendation. Two special activity groups also participated; these included older adult shopping-mall walkers and female trail users.

Each focus group discussion lasted 75 to 90 minutes. The moderator asked 17 scripted questions on product, price, place, and promotion and probed further as needed. The research staff constructed the questions with input from the community advisory council, which consisted of county residents with an interest in community health initiatives, after the council members were trained in social marketing strategies. The community advisory council training included an overview of the four principles of social marketing and discussions about the difference between commercial marketing and social marketing, audience segmentation, the role of research results in developing a marketing plan, and choosing a target audience. The Phase 1 and Phase 2 interview guides are available from http://prevention.sph.sc.edu/sips/PRPA_focusgroups.pdf.

As summarized in the Appendix, Phase 1 focus group questions assessed participants' perceptions of the product, its benefits and positioning, costs or pricing strategy, placement strategies, and promotional strategies in addition to their experiences, preferences, and attitudes concerning physical activity in general and walking in particular. During Phase 1, no specific duration or intensity of physical activity was described.

Two researchers independently assigned conceptual codes to sections of the transcribed text and met to reach consensus on coding categories. Analysis included both coding based on transcriptions from the focus groups and coding assisted by the NVivo (QSR International Pty Ltd, Victoria, Australia) software package. The results were categorized by the four Ps and by the discussion questions.

### Phase 2

The second phase consisted of five focus groups of 43 newly recruited women aged 35 to 54. Two of the groups were composed of regularly active women, and three of the groups were composed of irregularly active women. According to the anonymous questionnaire that the women completed, 58% of the 43 women interviewed were black and 40% were white. A description of Phase 2 focus group characteristics is shown in Table 2.

Phase 2 further defined the product (walking) and its benefits; it also identified possible strategies for promoting the product. Participants were first asked to distinguish between *physical activity *and* exercise *and to define* moderate activity*. As noted in the Appendix, women also were asked to share their opinions about using pedometers, to describe images that motivated them to be active, and to name or describe convincing spokespersons who would encourage them and their peers to be active. Finally, the women were asked about the radio and television stations they and their peers were most likely to listen to and how they would like to be contacted if they chose to participate in an exercise intervention. 

In Phase 2, two researchers also assigned conceptual codes independently to sections of transcribed text and met to reach consensus on coding categories. Because of the smaller number of focus groups in Phase 2 and the smaller number of transcripts compared with Phase 1, the use of analysis software to manage the transcription data was not essential. As in Phase 1, note-based analysis was used in Phase 2 following the same procedures for identifying and summarizing key themes.

## Results

### Phase 1: participant perceptions related to the principles of social marketing

On the basis of Phase 1 focus group findings, the community advisory council selected irregularly active women aged 35 to 54 as the target audience. They also identified the behavioral recommendation or actual product (4) as walking and other moderate-intensity physical activities for at least 30 minutes 5 or more days per week.

**Product and product positioning**

In this study, walking and gardening were the types of moderate-intensity physical activity preferred by participants in both phases of focus groups. The participants agreed, however, that walking should be the primary activity promoted in a social marketing program to increase physical activity because walking is easily accessible, low impact, and inexpensive. A few, principally those in regularly active groups, felt that walking should be just one of several aerobic activities promoted.

Participants cited several benefits of walking, including transportation, relieving stress, and spending time with family and friends, but all groups cited health as the primary reason to walk. Walking as a means of transportation was mentioned only in groups that included participants of lower SES; for example, participants who received public assistance or were in blue-collar positions. 

When asked about how they would motivate their friends to choose walking as a daily activity, participants responded with a variety of strategies that would position walking competitively with other preferred activities. Their responses suggest that walking may be positioned effectively by emphasizing body toning, social support opportunities, and decreased health risks that can result from regular physical activity. For example, having a walking companion may appeal to some women, thus combining social opportunities with exercise.

**Price**

The perceived price of walking varied among participants; however, most believed that their daily schedules were too full to include regular walking. In addition to time constraints, discomfort from the hot, humid climate and insects were commonly cited costs. 

**Place**

When participants were asked to describe their walking routine (i.e., when they walk, with whom, and what time of day), most preferred walking in the morning or early afternoon. All groups included a mix of participants who preferred walking alone or with someone. However, most female participants, regardless of age or activity level, preferred to walk with someone. Participants often cited their neighborhood as their favorite place to walk. The local shopping mall offered a walking program, but many focus group participants incorrectly assumed the program was for older adults only.

Sumter County has several public, multiuse trails and tracks, both paved and unpaved. Because the presence of trails has been shown to be associated with meeting the CDC's and American College of Sports Medicine's recommendation for physical activity (17), participants were asked about their perception of local trails. Their responses conflicted. Although most people appreciated trails for their abundance of foliage and tranquility, these same qualities led others to suspect that they were not safe or secure, especially for women. Some participants also found pet waste, litter, and inadequate lighting to be deterrents to using the trails.

**Promotion**

In this study, all focus groups highly favored walking groups, even though some individuals preferred walking alone. Many acknowledged that walking groups could provide walkers with social support, security, and childcare solutions. Regularly active men clearly stated that walking groups were appealing for women only. Unlike self-paced programs in which they could monitor their own progress using a pedometer, participants were less enthusiastic about community competitions.

Additionally, the participants suggested incentives that could be incorporated into a social marketing program. The incentives suggested varied with age and activity level. Regularly active participants preferred physical activity supports, such as athletic shoes, hats, or T-shirts; others (primarily irregularly active women) preferred monetary incentives or gift certificates.

### Phase 2: product refinement and promotional strategies

Based on Phase 1 focus groups and survey data, the community advisory council decided that the target audience would be irregularly active women aged 35 to 54. These women were attempting to be active but reported more barriers than men (e.g., responsibilities, lack of time). These women also found walking appealing and could influence their families and peers to walk. Because Phase 2 focus groups included the target audience, the discussion centered on refining the product for promotion.

When participants were asked to distinguish between *physical activity *and* exercise *and to define *moderate physical activity, *they described exercise as "structured activity" and physical activity as "unstructured activity," even incidental. Exercise, unlike physical activity, was described as "intentional," "purposeful," and "deliberate." Participants overwhelmingly preferred the term exercise to physical activity. Women held various perceptions of the term moderate activity. However, they all concurred that moderate physical activity requires exertion to be beneficial. 

Although most of the women in these groups had never before used a pedometer, they were willing to try one so that they could better monitor their activity. They found it especially appealing that pedometers could be used to set daily goals.

Participants from several groups stated that images of people exercising, particularly women, would motivate them to be active and expressed interest in the use of spokespersons. They strongly felt that the spokespersons should be women of various ages, races, and body shapes; they preferred spokeswomen with whom they could identify. Celebrities and well-known local women were mentioned as women who could motivate others to exercise. Local women recommended were women who were successful at losing weight or older women who were perceived to be active for their age.

As expected, the range of preferred radio and television stations was wide. The women admitted that they were most likely to listen to the radio when in the car, especially when traveling to and from work. When asked about how they would like to receive consistent feedback while participating in an intervention, almost all women felt that telephone calls were intrusive. Most preferred either e-mail or direct mail. Some women asked to receive tips that would encourage them to exercise.

## Discussion

The focus group findings were discussed among the community advisory council, the research team, and two professional advertising and publications consultants. The focus groups enriched our understanding of consumers' perceptions of walking and trail-use benefits and costs. Although walking was a favorite moderate-intensity exercise because it was considered to be an excellent way to improve physical and mental health, neither men nor women believed they had time to walk on a regular basis. Participants reported several priorities that dissuaded them from being active. Furthermore, even though Sumter County has several places where people can walk, some places, such as local trails, were misperceived as unsafe areas. Another misperception was that the local shopping-mall walking program was for older adults only.

### Use of focus group results to design a social marketing campaign

#### Product and product positioning

The focus group research enabled us to identify walking as the product to promote. Walking was perceived as a healthy activity for any age. However, the best way to position walking so it is competitive with other activities is to promote it as a way to spend time with friends and relatives and improve one's appearance while enjoying its well-known health benefits. The subsequent social marketing program focused on walking in an effort to provide an attractive and sustainable physical activity for women. 

#### Price

Although several costs were identified, lack of time was the greatest barrier for most participants. With further probing during focus group discussions, ideas for overcoming time limitations were elicited. For example, one could schedule walking time during the day as one would schedule a meeting at work. In an effort to make the time spent walking more acceptable, the social marketing program that was developed promoted walking as an opportunity to spend time with family and friends. 

#### Place

Most focus group participants clearly enjoyed walking in their neighborhoods, but some walking resources were unfamiliar to them. For example, the focus groups revealed the need to educate the community about the shopping mall walking program and local trail locations. In response to this need, the social marketing program highlighted a description of the shopping mall walking program and local trails in an exercise resource guide that lists places in the county to walk and be active. All of the women participating in the program received this guide.

An unanticipated finding was the overwhelming perception that local trails are potential sites for criminal activity. This finding was unexpected because according to the study's random-digit–dialed telephone survey, most people perceived trails as safe. Nevertheless, the program attempted to address safety concerns noted by the focus groups by organizing group walks on the trails. These group walks offered security to the women and provided an opportunity to meet potential walking partners.

#### Promotion

The focus group discussion centered on walking groups as a form of social support and security. Many participants, particularly women, favored self-paced programs with the use of a pedometer. Incentives were also identified; as a result of preferences expressed in the focus groups, the social marketing program incorporated a variety of incentives, including T-shirts, water bottles, and gift certificates. 

The Phase 2 focus groups, which consisted of irregularly active women aged 35 to 54, emphasized that marketing materials should incorporate images of women of diverse ages, body shapes, and races. It was clear that images of thin women were discouraging for this audience. In response to these findings, the resulting social marketing program invited local women of various ages, body shapes, and races to serve as program spokeswomen.

For this audience, marketing materials should perhaps include pedometers to motivate women to walk because the majority of women liked the idea of being able to measure their progress. All of the women who enrolled in the study received a pedometer and log to chart their progress in addition to receiving other educational materials.

Because most women participating in Phase 2 focus groups said they were more likely to listen to the radio while driving to and from work, the social marketing program used radio in addition to print media and television to promote walking and trail use and to recruit women for the exercise intervention. The spots were timed to coincide with the morning and afternoon commute.

Because telephone calls were seen as intrusive, participants received weekly tips on topics such as managing competing demands, goal setting, building social support, self-monitoring, and self-reward through their choice of mail or e-mail. In addition to those messages, the women received print materials, incentive prizes, and invitations to monthly exercise events with other women.

### Study limitations

There are caveats to this study. First, focus groups are not representative of an entire population, and participants' responses may be influenced by the responses of others. As a result, an issue such as crime on trails may be artificially inflated. Second, a focus group is not a tool for testing hypotheses; rather, it should be used to identify issues and themes. For these reasons, the study's focus groups were conducted in conjunction with quantitative data collection methods.

The essential components of social marketing — product, price, place, and promotion — must be considered and fully addressed for a social marketing program to be successful; this can only be done through consumer research. Because qualitative data are essential components of comprehensive consumer research, focus groups, as a qualitative research tool, can play a valuable role in enhancing consumer research. In this study, focus groups proved to be the key to understanding the target audience. As a result, a social marketing program promoting walking and other moderate-intensity physical activities was developed in Sumter County to meet the needs of irregularly active women.

## Figures and Tables

**Table 1:** Characteristics of Phase 1 Focus Group Participants for the Development of a Social Marketing Campaign, Sumter County, South Carolina

**Group**	**No.**	**Age, y**	**Sex (No.)**	**Race or Ethnicity (No.)**	**Special Interest Group or Socioeconomic Status (SES)**

**Regularly active ≥30 minutes per day, 5 or more days per week**					

1	5	29-65	Male (5)	Black (2) White (3)	Mixed SES
2	7	35-65	Female (7)	Black (4) White (3)	Mixed SES

**Irregularly active <30 minutes per day, 5 days per week**					

3	7	67-84	Male (3) Female (4)	Black (7)	Low SES
4	6	23-35	Male (6)	Black (6)	Low SES
5	8	18-44	Female (8)	Black (8)	Low SES caregivers
6	7	55-61	Female (7)	White (7)	Mid–high SES
7	7	44-72	Male (1) Female (6)	Black (4) White (2) Hispanic (1)	Mid–high SES
8	9	34-55	Male (9)	Black (3) White (6)	Mid–high SES
9	8	32-49	Female (8)	Black (7) Asian 1	Mid–high SES caregivers
10	10	36-51	Female (10)	Black (10)	Mid–high SES caregivers

**Special interest groups**					

11	10	60-86	3 Male 7 Female	Black 5 White 4 Unidentified 1	Shopping-mall walkers
12	6	31-63	6 Female	Black 1 White 5	Trail users

**Table 2:** Characteristics of Phase 2 Focus Group Participants (Women Aged 35 to 54) for the Development of a Social Marketing Campaign, Sumter County, South Carolina

**Group**	**No.**	**Race or Ethnicity (No.)**	**Socioeconomic Status (SES)**

**Regularly Active ≥30 Minutes per Day, 5 or More Days per Week**			

1	6	Black 2 White 4	Mixed SES
2	9	Black 2 White 7	Mixed–high SES

**Irregularly Active <30 Minutes per Day, 5 Days per Week**			

3	8	Black 7 White 1	Mixed SES
4	8	Black 3 White 5	Mixed SES
5	12	Black 1 Unidentified 1	Low SES

## Appendix. Findings From Phase 1 and Phase 2 Focus Group Responses and Related Program Components Used to Develop a Social Marketing Campaign in Sumter County, South Carolina

**Table:**

**Social Marketing Principle and Selected Question(s)**	**Salient Responses**	**Sample Participant Responses**	**Program Components**

**Product**			

*What types of physical activity do you like to do?*	Walking, gardening	"I like to walk because it gives me a chance to unwind and it relieves my stress and so I just walk." "I enjoy walking and gardening."	Educational messages, multiple media messages, and monthly exercise events promoted walking and other moderate-intensity physical activities.

**Product positioning**			

*What would you tell a friend to get him or her to take a walk regularly?*	Offer social support	"I like doing it, I'm going to keep calling one of my buddies, 'Hey, let's go girl, let's, you know, let's do it.' And we do it. You just got to keep one interested person."	Monthly exercise events (e.g., trail and neighborhood walks, low-impact aerobics, line-dancing classes) were scheduled so that women could meet each other and offer social support. Educational messages described ways that women could mobilize social support.

**Price**			

*What makes it difficult to walk regularly?*	Perceived lack of time	"…The problem with walking is it takes time. Probably to get as much good out of walking, you probably got to walk three times what you do running."	Educational messages and multiple media messages gave tips on how to fit exercise into a busy day, how to stay active while traveling, and how to include family members. Motivational messages encouraged women to walk on their lunch breaks, and a midday walk in the downtown business area was organized.
	Safety concerns	"In terms of someone hiding and attacking you because you are…vulnerable. …some parts of the trail are separated from society. …I mean it's public but it's secluded areas."	Educational messages and materials addressed safety, including walking with a partner and carrying a flashlight and wearing reflective clothing at night. Television spots depicted walking in groups.
	Competing responsibilities	"…I can't do it with my kids. I'll be too busy trying to focus on what they are doing. …when I get out and walk a lot of times that's just basically time for me…. Who's gonna keep my kids while I go to the track?"	Women could bring their children to the walking events. Multiple media messages addressed managing competing demands.
	Physical limitations	"I have spinal stenosis and …I have pain in the joints."	Educational materials and multiple media messages promoted various moderate-intensity physical activity options that could accommodate physical limitations. Educational messages and materials addressed the importance of stretching properly before and after exercise to prevent injuries.

**Place**			

*What might be appealing about walking on a trail?*	Animals and foliage Isolation and tranquility	"There's a lot of old-growth flowers and trees and bushes here and it's just very enjoyable to be out in." "…It's a shaded area and you're constantly looking, it's peace of mind, you're not bothered by other people or traffic. You're kind of isolated there. I like the environment…. a trail is peaceful."	An exercise resource guide included a list of trails, their locations, and hours of operation. Some monthly exercise events were held on various trails in the city and county. Multiple media messages highlighted trail walking as an exercise option and suggested ways to include family.
	Haven from automobile traffic and fumes	"It's just that you don't have to deal with traffic." "You don't have to deal with car fumes."	Several sections of trails were included in some of the monthly exercise events.
*What might be* unappealing *about walking on a trail?*	Trails are unsafe	"…I see a lot of men on the trails jogging. …I think sometimes they're not secure for a woman."	An exercise resource guide presented an extensive list of indoor and outdoor walking locations. Monthly events promoted use and visibility of trails.

**Promotion**			

*Would you participate in a walking club?*	Yes	"…You [could] have a parent to watch the children while some walk and then you rotate. I mean that way … somebody you trust… will take care of those kids and they walk the next [time]."	The research staff encouraged women who participated in the walks to exchange contact information so that walking buddies or teams could be formed. Monthly events were designed to facilitate development of social support. Educational messages promoted walking clubs.
	No	"I prefer to walk alone because of the pace…I need to do it by myself so I can get there and get back."	Educational messages, project spokeswomen (role models), and an exercise resource guide offered a variety of moderate physical activity options that could be done alone or with a partner or in a group.
*Would you participate in a community competition to promote walking?*	No	"It shouldn't be competition.…You exercise for your health not to compete." "I don't think that would be an incentive to anyone to walk. It may be an interesting study but I don't think it'd be an incentive to get people to walk."	Competition was not included because it was not appealing, but women were given a pedometer and an exercise log for self-monitoring.
*How are the words "physical activity" and "exercise"' different or the same from the word "exercise"?*	Physical activity is incidental, but exercise is deliberate	"Physical activity doesn't have enough *umph*!" "Physical activity is a form of exercise, but exercise is regular." "Exercise is planned."	The intervention used the term *exercise* as it implied planned, organized physical activity to the marketing audience and was the preferred term.
*When you hear the statement, "People should get at least 30 minutes of moderate activity 5 or more days per week," what does 'moderate activity' mean to you?*	Exertion is necessary for benefits	"The blood is flowing and the heart rate is elevated." "I think of walking."	Educational materials and multiple media messages included examples of moderate-intensity activities. The recommendation of 30 minutes on 5 or more days was a key theme of all materials and messages. Educational materials provided a guideline of 3500 to 4000 steps per 30 minutes as moderate-intensity walking.
*How would you feel about using a pedometer to track how much you are walking?*	Highly favored	"It's measurable and quantitative. Anything that is quantitative motivates me." "At the end of the day, you'd be surprised by how many steps you have taken during the day. I was doing more than what I thought I was actually doing. That's motivation."	All women were given a pedometer and asked to wear it daily to track their steps. The women used the pedometer to track their daily steps as well as the number of steps they accumulated during periods of exercise. They were given an exercise log to record their progress.
*Who would be a convincing spokesperson to encourage you and your women friends to walk or exercise?*	The spokespersons should be women who have diverse body shapes and skin tones and be of various ages. Should not appear thin	"Have more than one person — for different ages, weight levels — if you want to reach everybody." "I don't know of anyone specific, but it needs to be someone we can relate to — not a stick skinny person."	The program spokeswomen were recommended by focus group participants as active and admired women in the community. The spokeswomen varied in body shape.
*What kinds of images would be encouraging or motivating for women aged 35 to 54 to be more physically active?*	People exercising	"Have all different shapes, sizes, ages, colors because walking is for everybody."	The spokeswomen varied in age, race, weight, body shape, and occupation in addition to choice of activity.
